# High-Performance Gate-All-Around Field Effect Transistors Based on Orderly Arrays of Catalytic Si Nanowire Channels

**DOI:** 10.1007/s40820-025-01674-8

**Published:** 2025-02-19

**Authors:** Wei Liao, Wentao Qian, Junyang An, Lei Liang, Zhiyan Hu, Junzhuan Wang, Linwei Yu

**Affiliations:** School of Electronic Science & Engineering, Nanjing University, Nanjing, 210093 People’s Republic of China

**Keywords:** In-plane solid-liquid-solid, Ultrathin silicon nanowires, Gate-all-around field-effect transistors (GAA-FETs)

## Abstract

Gate-all-around field-effect transistors (GAA-FETs) represent the leading-edge channel architecture for constructing state-of-the-art high-performance FETs. Despite the advantages offered by the GAA configuration, its application to catalytic silicon nanowire (SiNW) channels, known for facile low-temperature fabrication and high yield, has faced challenges primarily due to issues with precise positioning and alignment. In exploring this promising avenue, we employed an in-plane solid–liquid-solid (IPSLS) growth technique to batch-fabricate orderly arrays of ultrathin SiNWs, with diameters of *D*_NW_ = 22.4 ± 2.4 nm and interwire spacing of 90 nm. An in situ channel-releasing technique has been developed to well preserve the geometry integrity of suspended SiNW arrays. By optimizing the source/drain contacts, high-performance GAA-FET devices have been successfully fabricated, based on these catalytic SiNW channels for the first time, yielding a high on/off current ratio of 10^7^ and a steep subthreshold swing of 66 mV dec^−1^, closing the performance gap between the catalytic SiNW-FETs and state-of-the-art GAA-FETs fabricated by using advanced top-down EBL and EUV lithography. These results indicate that catalytic IPSLS SiNWs can also serve as the ideal 1D channels for scalable fabrication of high-performance GAA-FETs, well suited for monolithic 3D integrations.

**Supplementary Information:**

The online version contains supplementary material available at 10.1007/s40820-025-01674-8.

## Highlights

- A high-density array of orderly silicon nanowires (SiNWs) was grown in precise locations, with diameter of *D*_NW_ = 22.4 ± 2.4 nm and interwire spacing of 90 nm.
- A special suspension-contact protocol has been developed to reliably suspend the in-plane solid-liquid-solid SiNWs to serve as ultrathin quasi-1D channels for gate-all-around field-effect transistors (GAA-FETs).
- By optimizing the source/drain metal contacts, high-performance catalytical GAA-FETs have been successfully demonstrated, achieving a high on/off current ratio of 10^7^ and a steep subthreshold swing of 66 mV dec^-1^.

**Supplementary Information:**

The online version contains supplementary material available at 10.1007/s40820-025-01674-8.

## Graphical abstract

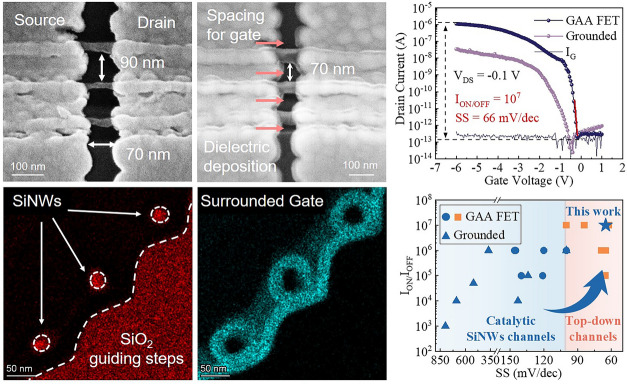

## Introduction

**Fig. 1:**
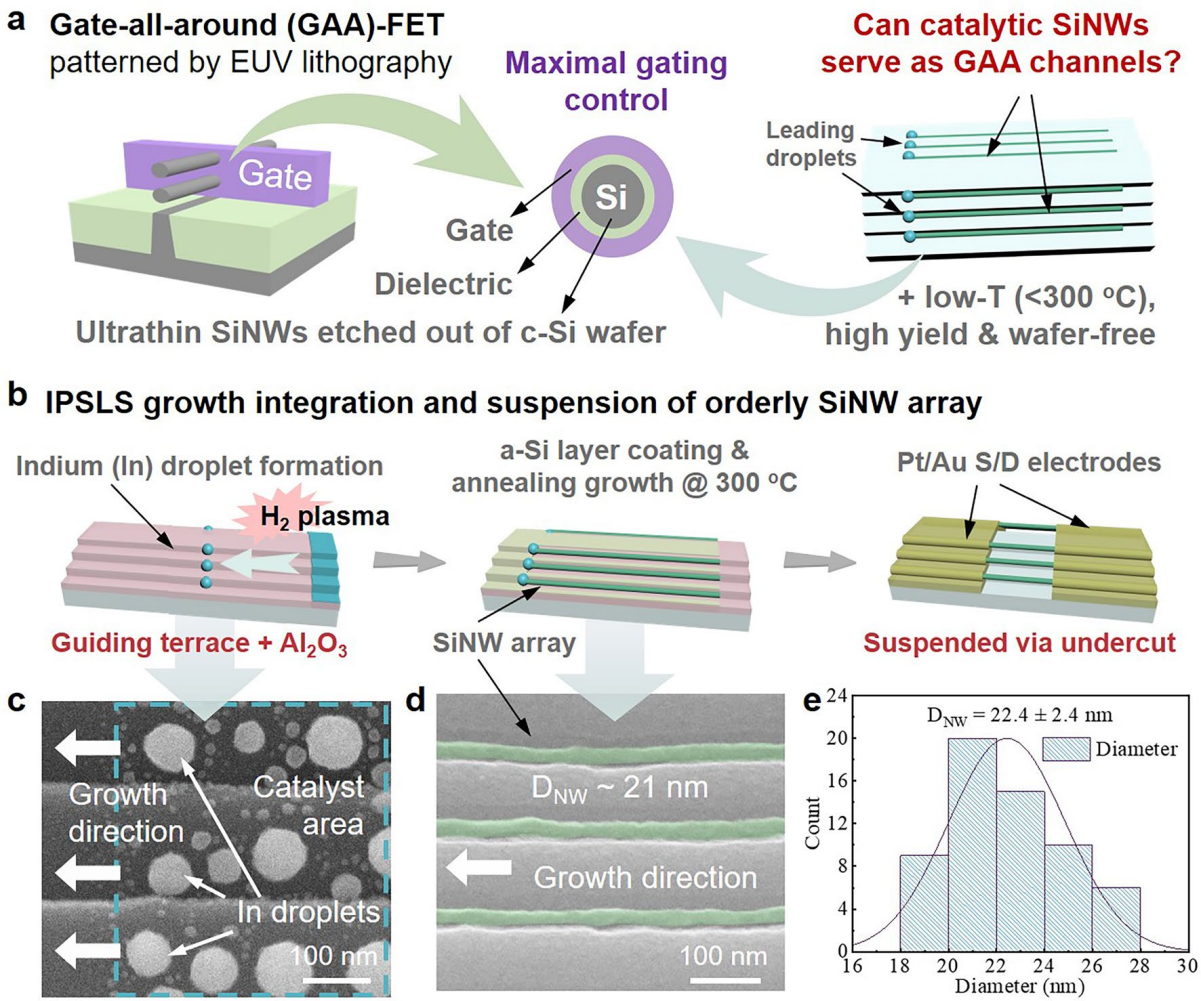
**a** Schematic illustration of GAA structure fabricated via EUV lithography and etching and catalytic growth integration, **b** IPSLS growth integration and suspension of orderly SiNW array, typical SEM images of **c** the catalyst area and **d** as-grown SiNWs, **e** statistics on the diameter of as-grown ultrathin IPSLS SiNWs

Gate-all-around field-effect transistors (GAA-FETs, as depicted in Fig. 1a), which offer exceptional electrostatic control, are becoming the mainstream device architecture for technology nodes < N3 nm [1–4], where ultrathin crystalline silicon nanowires (c-SiNWs) are considered as ideal quasi-one-dimensional (1D) channel materials to mitigate the short-channel effect in highly integrated CMOS logics. However, to implement monolithic 3D integration that enables higher integration density, it is essential to fabricate ultrathin SiNW channels on the stacked layers, where a monocrystalline Si wafer substrate is absent. Instead of using a conventional top-down etching procedure in the bottom logic layer, these 1D SiNW channels should be grown in precise locations as orderly arrays directly on the insulating dielectric layer, via a low-temperature process to avoid thermal damage to the underlying logic layers [5–7].

To address this challenge, a low-temperature catalytic growth of such SiNW channels provides an ideal option, as it doesn’t rely on the preexistence of c-Si wafer [8, 9]. Although the famous vapor–liquid-solid (VLS) growth mechanism has been widely exploited to produce high-quality SiNW channels for the demonstration of high-performance FETs and sensors [10–13], the vertical orientation of the VLS-grown SiNWs makes them difficult to integrate into the planar device architecture [14, 15]. Particularly, the post-growth transfer and release of individual VLS-SiNWs onto pre-defined electrode trench, for the subsequent formation of GAA-FET, lacks deterministic position and orientation control, and is thus technically incompatible with the standard planar manufacturing procedure [11, 16, 17]. In order to address this challenge, an in-plane solid–liquid-solid (IPSLS) mechanism [18–20] has been developed in our previous works, where indium (In) catalyst droplets absorb amorphous silicon (a-Si) precursor thin film to produce c-SiNWs along pre-defined guiding step edges. Indeed, a rather high-density integration of the IPSLS SiNWs has been demonstrated, under the guidance of terrace mini-steps [21, 22] or the sidewall grooves [23, 24], achieving an impressive diameter and uniformity control, backed by a series of tailored catalyst formation technologies [24, 25]. However, the performance of planar or GAA-FETs fabricated with catalytic SiNW channels still lags significantly behind that of state-of-the-art fin- or GAA gate FETs [11, 16, 17, 26]. Specifically, the subthreshold swing (SS) of the catalytic SiNW-FETs, a key indicator of the strength of electrostatic control, typically ranges from 850 to 100 mV dec^−1^ [27–33], which is still far from the theoretical limit of 60 mV dec^−1^. For the IPSLS SiNWs, a genuine GAA-FET has not yet been demonstrated, and their potential to serve as 1D channels for high-performance FETs remains to be explored and verified through direct experimental evidence.

In this work, we focus on demonstrating the first-ever GAA-FET based on catalytic IPSLS SiNWs, by developing a series of critical channel-releasing and contacting techniques. First, ultrathin SiNWs with diameter of D_NW_ = 22.4 ± 2.4 nm and interwire spacing of 90 nm were grown on a pre-designed sacrificial layer. Then, a specialized suspension-contact protocol was developed to reliably release these ultrathin channels, achieving suspended SiNWs with a maximal suspension length exceeding 500 nm. By optimizing the source/drain metal contacts, high-performance junctionless GAA-FETs have been successfully demonstrated, exhibiting an *I*_on/off_ ratio of 10^7^ and a sharp SS of 66 mV dec^−1^. These results represent the first experimental evidence that catalytic grown SiNWs can also serve as high-quality channels for the fabrication of GAA-FETs, achieving high-performance comparable to those fabricated using sophisticated top-down EBL and EUV lithography methods.

## Results and Discussion

**Fig. 2:**
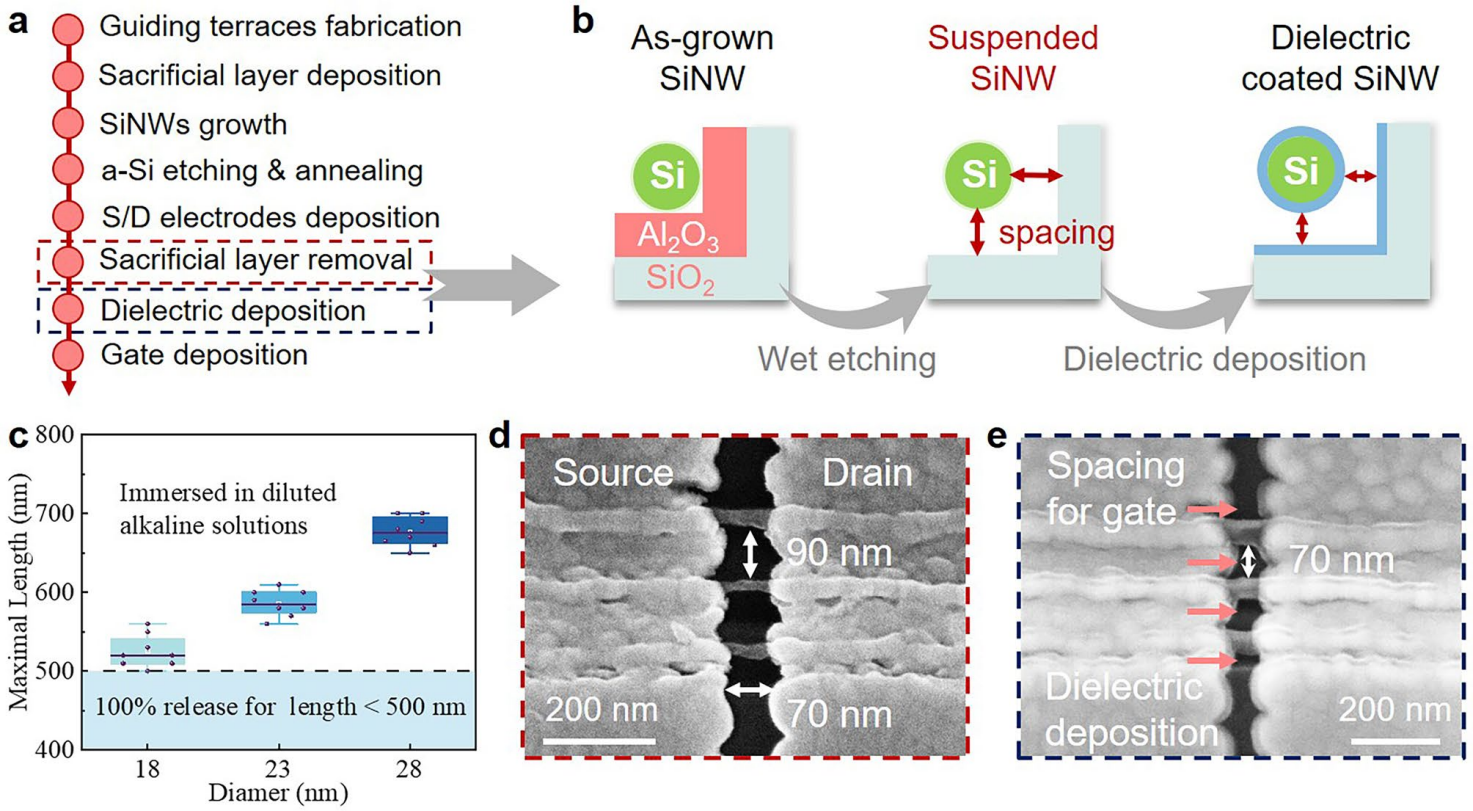
**a** Fabrication processes of SiNW GAA-FETs, **b** schematic illustrations of the undercut-releasing and dielectric deposition steps. **c** Statistics on the maximum suspension length for the SiNW channels of different diameters. **d**–**e** Typical SEM images of the suspended SiNWs, held by S/D electrode pads, before and after dielectric deposition of 3 nm Al_2_O_3_ and 7 nm HfO_2_

The SiNWs were grown upon 500 nm oxide-coated c-Si wafer substrate, via IPSLS mechanism as depicted schematically in Fig. 1b. Specifically, the guiding terrace edges were first patterned by using lithography, followed by multiple alternating cycles of C_4_F_8_ and O_2_ plasma etching processes in inductively coupled plasma (ICP) system, to form a multi-step guiding terraces, as specified in the Supporting Information. Subsequently, a 40-nm layer of Al_2_O_3_ was deposited as the sacrificial layer using atomic layer deposition (ALD). Then, as depicted schematically in the three panels of Fig. 1b, the IPSLS growth of SiNWs involves two major steps that is (1) the patterning and deposition of indium (In) catalyst stripes, of nominally 4 nm thick, at the starting ends of the guiding terraces. After loading into a PECVD system and being treated by H_2_ plasma at 215 °C, the In stripes were transformed into discrete droplets with diameters of 70 ± 11 nm, as witnessed in the scanning electron microscopy (SEM) image in Fig. 1c; (2) the deposition of 7 nm thick a-Si precursor film over the whole sample surface at a lower temperature at 110 °C (below the In melting point), followed by annealing in vacuum after raising the substrate temperature to 290 °C, which activated the droplets to absorb the a-Si layer, move along the terrace steps, and produce ultrathin SiNWs. At the end, the source/drain (S/D) electrode pads, of a bilayer of platinum (Pt)/gold (Au) of 12/55 nm thick, were patterned by using electron beam lithography (EBL) and deposited by electron beam evaporation.

In order to release the SiNWs to form suspended channels, the exposed sacrificial Al_2_O_3_ layer, uncovered by the S/D pads, was etched off by immersing in diluted alkaline solutions (2.5%), and more experimental details are provided in the Supporting Information. According to a typical SEM image of the as-grown SiNWs presented in Fig. 1d, the parallel SiNWs, pseudo-colored in green for the sake of clarity, show a uniform diameter of approximately 21 nm and are well positioned at the roots of the mini-steps of the guiding terrace. Note that, a high uniformity of the as-grown SiNWs, with an average diameter of* D*_NW_= 22.4 ± 2.4 nm, has been achieved here mainly through the In thickness control and subsequent droplets formation [22], as witnessed in the statistics shown in Fig. 1e.

Figure 2a summarizes the whole fabrication procedure, where the key steps of forming suspended SiNW channels that is the undercut-releasing and dielectric deposition steps, have been highlighted by two dash rectangles. Specifically, a rather conformal Al_2_O_3_ sacrificial layer of 40 nm thick has been coated on the terrace surface, by using ALD, which can be safely removed by immersing in a diluted alkaline solution, as depicted in Fig. 2b, leaving a suspended array of SiNW channels held by the S/D metal pads at the two ends. Despite such a simple solution etching-releasing procedure, without using a supercritical releasing technique [34], this Al_2_O_3_-alkaline undercutting has proven to be gentle enough to presume the geometry and integrity of the suspended SiNWs. For example, Fig. S1 presents the SEM images of the well-released and suspended SiNWs with spanning lengths of 100, 250, 310, 460, and even up to 700 nm, respectively. For even longer suspension lengths, the SiNWs were found to either attach to the substrate or break, being dragged down by the retracting liquid interfacial force as they were pulled out of the etching solution.

The maximum suspension length is also correlated with the diameter of SiNW, as evidenced in the statistics presented in Fig. 2c. For example, SiNWs with the largest diameter of 28 nm can be successfully released to form suspended channels throughout 700 nm with a 100% survival rate. In contrast, the thinnest SiNWs, with diameters of 18 nm, can only be safely released over a suspension length of less than 500 nm. However, even this shorter length is sufficient for suspending the SiNWs to serve as GAA-FET channels. After undercut-releasing three parallel SiNWs as channels, to form a suspended channel length of *L*_*nw*_ ~ 70 nm, with a close interwire spacing of 90 nm, as seen in Fig. 2d, a uniform stacked dielectric layer of 3 nm Al_2_O_3_ and 7 nm HfO_2_ was deposited by using ALD (Fig. 2e). It is important to note that the SiNWs are now suspended at a distance of 40 nm from both the substrate and the sidewall, determined by the thickness of the sacrificial Al_2_O_3_ layer. This separation provides sufficient space for applying the dielectric deposition and the subsequent wrapping of the gate metal, which is a critical requirement for fabricating a genuine gate-all-around (GAA) FET device.

**Fig. 3:**
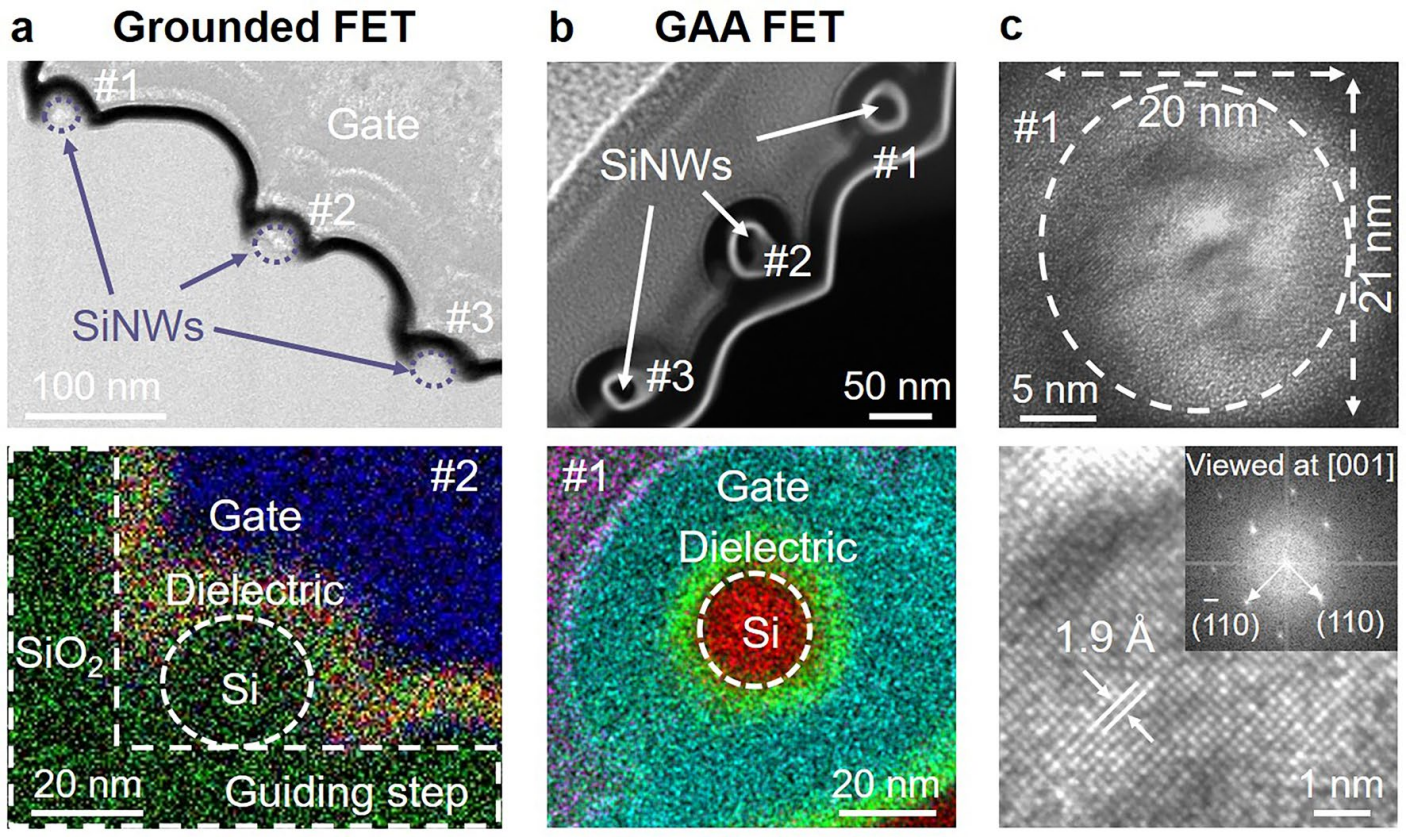
Cross-section TEM images and corresponding high-resolution EDS maps of the IPSLS SiNW-FETs: **a** Grounded and **b** GAA-FET. **c** Enlarged HR-TEM images and corresponding electron diffraction pattern of #1 SiNWs in GAA-FET

After the dielectric layer deposition step, the suspended SiNW channels were covered with the gating electrode, resulting in a typical GAA configuration. In comparison, a reference FET with grounded SiNW channels, unreleased ones resided at the roots of step corners, was also prepared. Figure 3a, b shows the cross-sectional high-resolution transmission electron microscopy (HR-TEM) analysis of the grounded and released (GAA) SiNW channels, respectively. It is important to note that, according to the detailed SEM inspections and EDS (energy-dispersive spectroscopy) mapping of the exposed channel cross sections, the grounded SiNW channels (seen in Fig. 3a) can only be effectively gated by the gate electrode from the top and the right sides, limited geometrically by the concave step corner sidewalls. In contrast, in a GAA configuration, as shown in Fig. 3b, the SiNW channels can be fully wrapped around and gated in all directions to achieve the maximum capacitive gate-channel coupling. Furthermore, as illustrated in Fig. 3c, the enlarged HR-TEM image reveals a slightly ellipsoidal SiNW, with a height and width of approximately 20 and 21 nm, respectively. This particular SiNW has been found to be monocrystalline with a clear lattice fringe spacing of 0.19 nm, corresponding to that of the Si (110) planes. Meanwhile, the fast Fourier transform (FFT) pattern displayed in the inset indicates that the growth direction of this specific SiNW is along Si < 100 > , while Si < 110 > is more commonly observed according to the statistics of growth orientations in Fig. S2, as well as those shown in our previous works [23, 24].

**Fig. 4:**
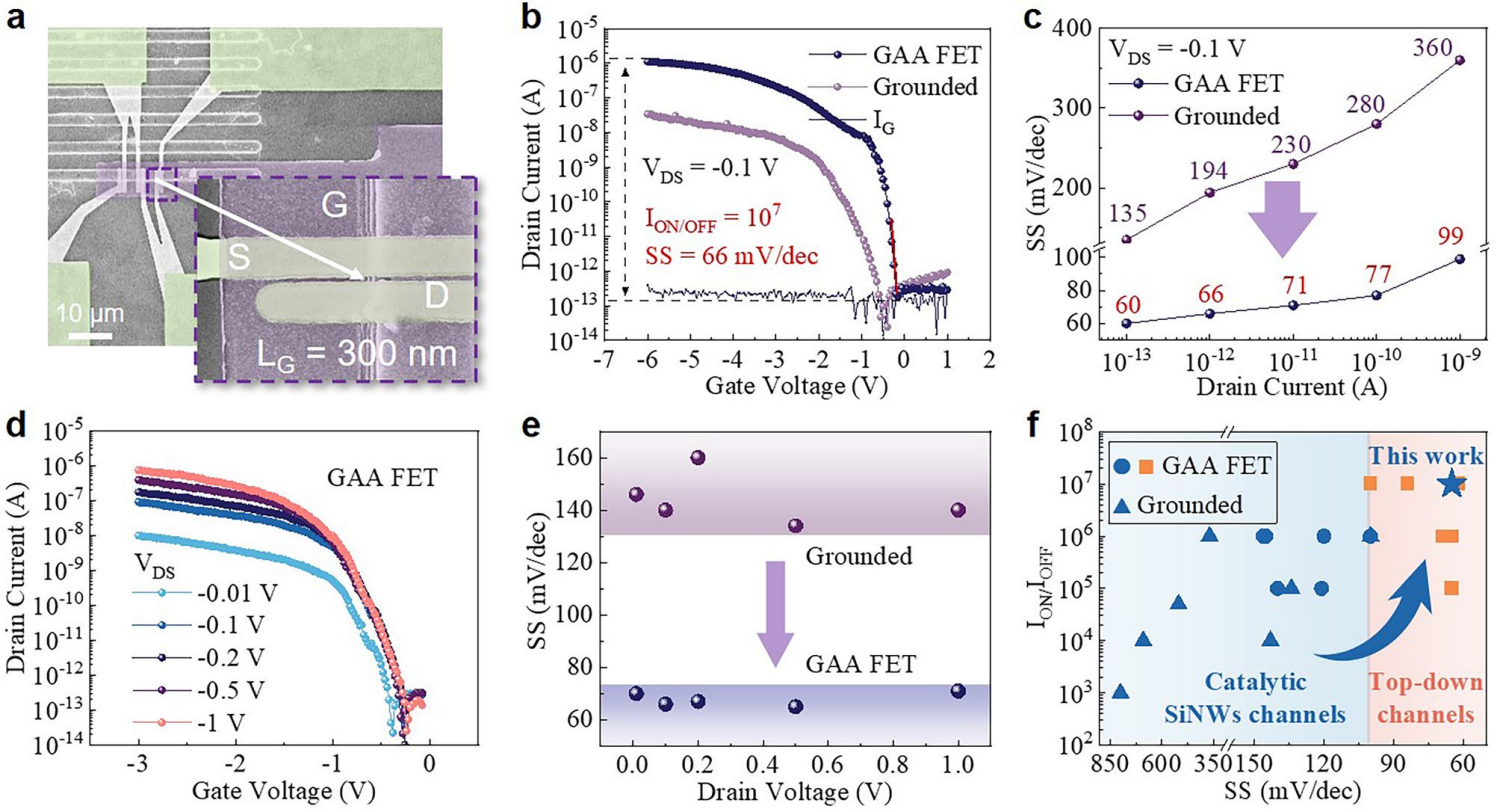
**a** SEM images of the GAA-FET. **b** *I*_*D*_* − V*_*G*_ curves (V_D_ = 0.1 V) and **c** *SS − I*_*D*_ curves of grounded FET and GAA-FET. **d** Typical transfer curves of GAA-FET. **e** *SS − V*_*D*_ curves of grounded FET and GAA-FET. **f** Comparison of the SS and *I*_ON_/*I*_OFF_ ratio of FETs based on catalytic SiNW [10, 11, 16, 17, 27–33, 40] and top-down etched SiNW/NS [26, 36–38, 41, 42] in the literature

Figure 4a presents an SEM image of the as-fabricated GAA-FET, with a channel length of 300 nm and a pair of source/drain (S/D) contact electrodes of Pt (12 nm)/Au (55 nm). As illustrated in Fig. 4b, under a bias of *V*_DS_ = − 0.1 V, the GAA-FET demonstrates a high *I*_ON_/*I*_OFF_ ratio of 10^7^, a sharp SS of 66 mV dec^−1^ and a low leakage current < 0.1 pA (corresponding to an off-state power consumption < 0.03 pW and a conductive power consumption of ~ 0.2 μW). In comparison, the reference FET with grounded SiNW channels exhibits an *I*_ON_/*I*_OFF_ ratio of < 10^6^ and a higher SS = 150 mV dec^−1^. More systematic comparison of the SS performance evolution trends of the GAA- or grounded FETs, at different channel current loads and biases (as depicted in Figs. 4d and S3), are staged in Fig. 4c, e, respectively. It is found that the GAA configuration can indeed largely enhance the field-effect gating efficiency of the SiNW-FET, achieving a much lower SS approaching the theoretical limit of 60 mV dec^−1^ [35]. In addition, the transfer characteristics of 20 randomly selected SiNW GAA-FETs are depicted in Fig. S4, where all devices show an average *I*_on/off_ current ratio > 10^6^ under a bias of *V*_DS_ = − 0.1 V. The SS values, ranging from 64 to 85 mV dec^−1^, are also comparable to those observed in the cutting-edge top-down GAA-FET devices [26, 36–38].

This finding also emphasizes the fact that a more efficient gating configuration is required to fulfill the potential of the catalytic SiNWs to serve as the 1D channels for high-performance FETs. One of the major reasons for the insufficient gating control of the SiNW can be assigned to the relatively high indium (In) atom density incorporated into the SiNWs during the IPSLS growth, which is known to introduce *p*-type doping in c-Si and has been verified to be *n*_*In*_ ~ 10^19^ cm^−3^ in the as-grown SiNWs in our previous works [33, 39]. Though the dissolved In atom density can be substantially reduced via a post-growth annealing to below 10^16^ cm^−3^, a more straight strategy is to reduce the diameter of the SiNW channel, to be smaller than the Debye screening length of the In-doped SiNWs, *D*_nw_ < *λ*, where ${\uplambda } \sim {\text{n}}_{{{\text{In}}}}^{{ - 1/2}}$ is in the order of ~ 10 nm for a doping concentration of 10^16^ cm^−3^. However, in the GAA-FET configuration, the field-effect gating can be applied from all directions, and thus this Debye screening length restriction can be extended to *D*_nw_*/2* < *λ*. Or, in other words, the carrier density in SiNW can be more effectively modulated to achieve a far more efficient channel current switching control, to achieve a lower SS ~ 60 mV dec^−1^, approaching the theoretical limit.

**Fig. 5:**
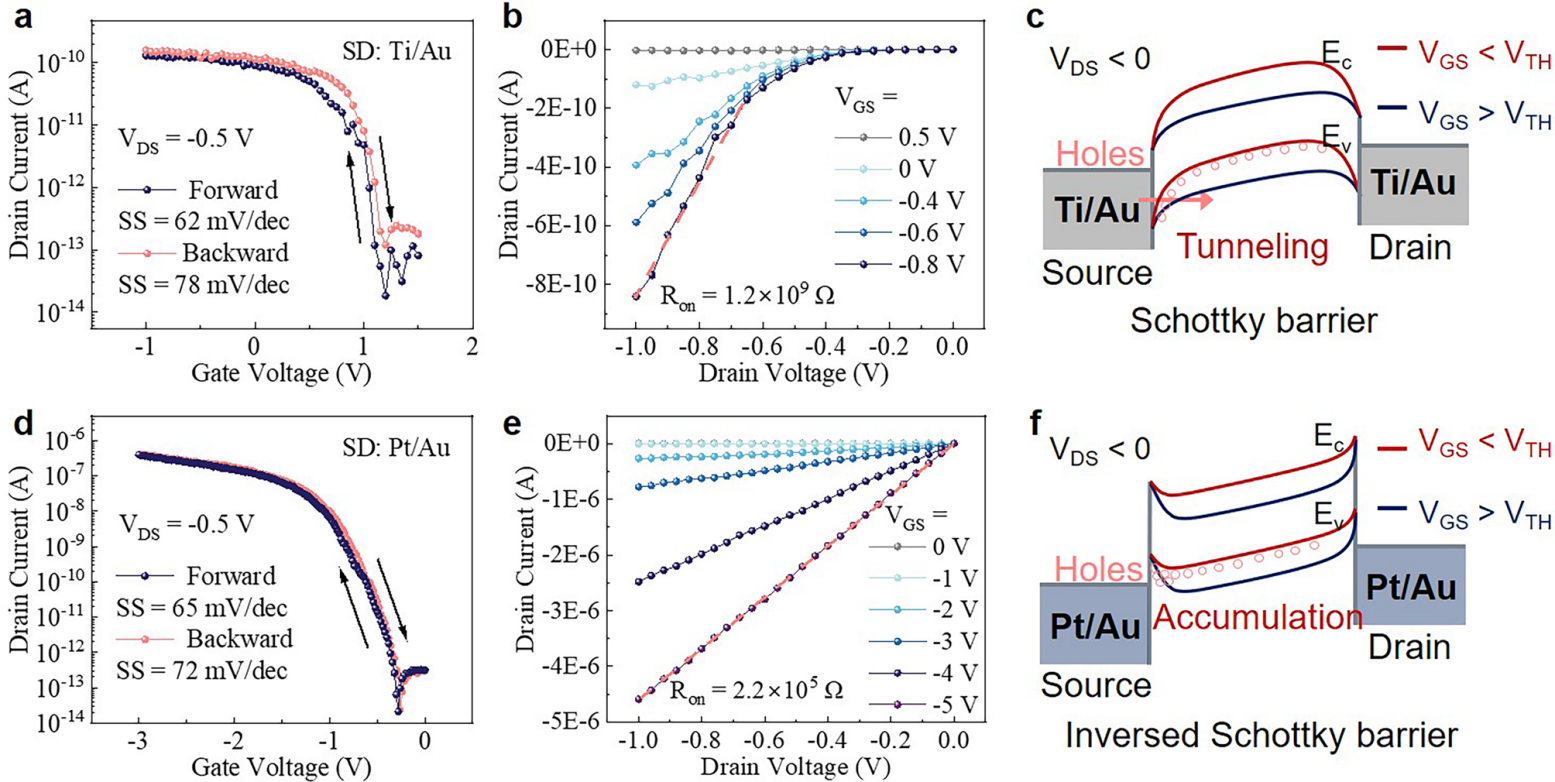
**a** Hysteresis curves and **b** output curves of the Ti/Au contacted GAA-FET, **c** corresponding band gap alignment profiles under different gating bias conditions, where a large Schottky barrier is formed at the Ti/SiNW contact, **d** hysteresis curves and **e** output curves of the Pt/Au contacted GAA-FET, **f** corresponding band gap alignment profiles under different gating bias conditions, where the Pt/SiNW contact forms an inverse Schottky barrier

On the other hand, it is also important to note that, there is no doping in the S/D contact regions and SiNW-FETs demonstrated here are in a convenient junctionless configuration. So, the choice of suitable S/D contact metals, with the right work function that matches the doping style and concentration in the SiNW channels, is another key control parameter to obtain a lower contact resistance to boost the drive current. Here, GAA-FETs with Ti/Au (12/55 nm) or Pt/Au (12/55 nm) as S/D contact electrodes were fabricated and compared. As shown in the typical transfer curves shown in Fig. 5a–d, the Ti/Au contacted GAA device delivers a lower on–off current ratio of only 10^3^, despite a sharp SS of 62 mV dec^−1^ has been recorded at the early turning-on regime. In comparison, with basically identical GAA channel conditions, the Pt/Au contacted GAA-FET exhibits a remarkable increase in on current by nearly four orders of magnitude, achieving simultaneously high *I*_on/off_ = 10^7^ and an SS = 65 mV dec^−1^. Note that, both of the GAA-FETs demonstrate a negligible hysteresis in forward and backward sweeps, but with slightly higher SS in backward track.

According to the output characteristics presented in Fig. 5b–e, it is evident that the Pt/Au contacted SiNW-FETs behave more like Ohmic contact than the Ti/Au contacted ones. Specifically, the on-state resistance (*R*_on_) of the Pt/Au contacted SiNW-FET (2.2 × 10^5^ Ω) is reduced by more than three orders of magnitude relative to that of the Ti/Au contacted device (1.2 × 10^9^ Ω). This phenomenon can be attributed to the different work functions of the primary contact metals of Ti and Pt. As illustrated in the energy band diagrams of Fig. 5c, f, a work function of Ti is about 4.3 eV, 0.2 eV higher than the valence band top of c-Si, and thus will tend to pull up the band profile of c-SiNW channel to create a large Schottky barrier at the Ti/SiNW contact. To turn on the SiNW channel connected by a pair of back-to-back Schottky barriers, a negative gating voltage of *V*_GS_ < *V*_TH_ is required to force the triangle Schottky barrier to become thinner and thinner, to allow for direct hole tunneling. However, the existence of such Schottky barriers poses a fundamental limit for the transport current, particularly in high current on state, as evidenced in Fig. 5a. So, by choosing a metal with deeper work function of approximately 5.6 eV for Pt, this situation can be greatly facilitated. As diagrammed in Fig. 5f, the Pt/p-SiNW contact forms an inverse Schottky barrier, and thus makes it possible to boost the on current of the GAA-FETs.

As summarized in Fig. 4f, although catalytic SiNWs have been widely investigated as promising channel materials for high-performance FETs over the last two decades, their subthreshold swing (SS) performances have typically been limited to a range of 850–100 mV dec^−1^ [27–33]. Among them, SiNW-FETs with GAA gating have achieved, on average, a higher on/off ratio up to 10^6^ and a smaller SS approaching 100 mV dec^−1^ [10, 11, 16, 17, 40], as represented by the filled blue circles. This limit has now been broken in this work, with the ultrathin SiNW GAA-FETs denoted by a blue star, which stand in the upper-right corner of the high-performance region in Fig. 4f (with *I*_on/off_ > 10^7^ and SS < 90 mV dec^−1^). This region has long been occupied only by the cutting-edge fin or GAA gate FETs marked by the orange rectangles [26, 36–38, 41, 42].

This comparison can be better understood with more technical details, as provided in Table 1. The catalytic SiNWs, grown primarily through the most popular VLS mechanism, typically exhibit a larger diameter and less stringent uniformity control, usually ranging from 30 to 150 nm. The relatively thicker diameter and larger diameter variation of the VLS-grown SiNWs are major reasons for the poorer channel current modulation efficiency, and thus higher SS of the VLS-SiNW-FETs, even fabricated in a GAA gating configuration. More importantly, vertically grown VLS SiNWs are difficult to integrate into the mainstream planar device architecture, and thus necessitating a complex post-transfer and re-arrangement of the tiny individual SiNWs for the subsequent fabrication of GAA-FETs. On the other hand, in contrast to the standard top-down fin or GAA gate FETs, most of the SiNW-FETs, including those in this work, are fabricated with a junctionless S/D metal contact. This thus requires a careful choice of suitable S/D contact metals to match the channel doping polarity in the SiNW channels. Otherwise, the formation of a pair of Schottky barriers at the S/D contact will seriously degrade the electronic transport performance, as already evidenced here.

**Table 1:** Comparison of our GAA-FET to the SiNW/NS GAA-FETs in other literature

Fabrication strategies		D_NW_ or CD* (nm)	I_ON_ (A)	*I*_ON_/*I*_OFF_	|V_D_| (V)	SS (mV dec^−1^)	References
Top-down etching	EBL	7–18	10^2^	10^5^	1	140	[41]
	EBL	8	10^−6^	10^7^	0.1	62	[36]
	EBL	8–10	10^−5^	10^7^	1	84	[37]
	Edge mask	6	10^−6^	10^5^	0.9	65.1	[38]
	Edge mask	8	10^−4^	10^6^	0.7	69.2	[42]
	EUV lithography	4	10^−6^	10^6^	0.65	65	[26]
Catalytic growth	VLS (V-FET)	20–30	10^−7^	10^6^	0.25	120	[10]
	VLS and transfer	25–60	10^−7^	10^6^	0.5	105	[11]
	VLS and transfer	20–120	10^−8^	10^5^	1	121	[16]
	VLS and transfer	100–150	10^−6^	10^6^	0.95	145	[17]
	VLS over trench	120–150	10^−7^	10^6^	0.05	146	[40]
	IPSLS	22.4 ± 2.4	10^−6^	10^7^	0.1	66	This work

*Critical dimension: the smallest thickness/width of nanosheet channels

With that said, the role of catalytic SiNWs in the development of high-performance GAA-FETs should not be discounted. On the contrary, our results provide straightforward experimental evidence that, with ultrathin and uniform diameter control and appropriate S/D contact, GAA-FETs built upon catalytic SiNW can also achieve excellent FET performance comparable to those fabricated with cutting-edge top-down lithography technology. Compared to the traditional GAA-FET fabrication that relies on sophisticated EUV/EBL lithography to pattern/define ultrathin c-Si channels (< 20 nm) out of Si/SiGe superlattice multilayers deposited on c-Si wafer, these catalytic SiNWs can be grown as orderly arrays at low temperatures (< 300 °C) upon dielectric substrate, without the need of the pre-existing c-Si wafer and the use of high-precision lithography [24]. All these unique features of IPSLS catalytic SiNWs make them particularly suited for constructing a new generation of 3D integrated electronics or computing-in-memory applications. Compared to the commonly used channel materials for monolithic 3D integrations, such as amorphous oxide semiconductor (AOS), low-dimensional materials, and poly-Si-based FETs reported in the literature and summarized in Table S1, the SiNW GAA-FET also demonstrates a superior performance in terms of SS and on–off current ratio [43–53].

However, it is equally important to point out that the catalytic SiNW channels still need to improve in terms of size and lattice uniformity, compared to the stringent parameter control achieved in standard top-down etched GAA channels. Also, the SiNW array fabricated here is not arranged in a high-density stacked manner as in the standard GAA-FET configuration [26, 38, 42]. So, a sidewall groove-guided growth integration of even thinner SiNWs, as demonstrated in our previous works [23, 24], still needs to be testified to improve further the channel integration density. Moreover, the contact resistance under the metal-SiNW S/D electrode also needs to be reduced by suitable doping control or ion-implantation technique. To this end, the a-Si precursor pre-doping control strategy, established in our previous works [54], can help to adjust the effective channel doping and ameliorate the S/D contact. Finally, a complementary n-type GAA-FET has yet to be demonstrated in our future work for building high-density CMOS logic with reduced contacted gate pitch.

## Conclusion

In summary, we have demonstrated high-performance GAA-FETs based on catalytic IPSLS SiNWs, which can be grown into orderly arrays, with ultrathin diameters of *D*_NW_ = 22.4 ± 2.4 nm and close interwire spacing of 90 nm. A series of undercut-releasing and optimized S/D contact techniques have been successfully developed to establish reliable and scalable fabrication protocols of GAA-FET logic. For the first time, the electronic transport performance of junctionless FETs with catalytic SiNW channels has been largely improved, achieving an excellent *I*_on/off_ current ratio of 10^7^ and a steep SS of 66 mV dec^−1^, comparable to those fabricated with the aid of top-down EBL and EUV lithography. These results represent the first experimental evidence that the catalytic SiNWs grown via a low-temperature IPSLS process hold important potential for building state-of-the-art high-performance GAA-FETs, particularly suited for monolithic 3D integrations.

## Supplementary Information

Below is the link to the electronic supplementary material.

Supplementary file1 (DOCX 1568 kb)
