# Supplementary material for: High-Performance Gate-All-Around Field Effect Transistors Based on Orderly Arrays of Catalytic Si Nanowire Channels
Source: Nanomicro Lett. 2025 Feb 19;17:154. doi: 10.1007/s40820-025-01674-8 (PMC11839962; doi:10.1007/s40820-025-01674-8)
Supplement: Supplementary file 1 — Supplementary file1 (DOCX 1568 kb) [file 40820_2025_1674_MOESM1_ESM.docx]

Supporting Information for

**High-Performance Gate-All-Around Field Effect Transistors Based on Orderly Arrays of Catalytic Si Nanowire Channels**

Wei Liao^1^, Wentao Qian^1^, Junyang An^1^, Lei Liang^1^, Zhiyan Hu^1^, Junzhuan Wang^1^, Linwei Yu^1*^

^1^School of Electronic Science & Engineering, Nanjing University, Nanjing 210093, P. R. China

*Corresponding author. E-mail: yulinwei@nju.edu.cn (Linwei Yu)

# S1 Experimental Section

***Guiding terraces fabrication****:* A commercial wafer, featuring a 500 nm oxide layer, was subjected to an ultrasonic cleaning sequence with N-methylpyrrolidone, ethanol, and deionized water. Photoresistor stripes of AZ5214, 3 µm wide and 1.5 µm thick, were then patterned on the substrates via photolithography. This was succeeded by 3 times alternating ICP etching processes, where single time recipe included C_4_F_8_ plasma etching (12.5 sccm, 1.8 mTorr, 30 W for 30 s) and O_2_ plasma etching (30 sccm, 30 mTorr, 65 W for 75 s). Here, the width of the guiding terraces can be well controlled by adjusting the parameters of O_2_ plasma etching (duration/power), achieving an interwire spacing of <100 nm. Then, the etched substrate concluded sequentially with an 8-minute ultrasonic cleaning cycle using N-methylpyrrolidone, ethanol, and deionized water. Finally, a Al_2_O_3_ sacrificial layer of 40 nm thick has been coated on the terrace surface, by using atomic layer deposition (ALD).

***Catalyst deposition and SiNWs growth****:* The In catalyst region was patterned on the above substrate using standard photo-lithography. A 4 nm thick In layer was subsequently deposited via thermal evaporation, followed by a standard lift-off process to remove residual photoresist and In. The sample was then transferred into the PECVD system for SiNW growth. Firstly, a H_2_ plasma treatment at 215 °C for 4 minutes was performed with a H_2_ flow rate of 14.8 sccm and a chamber pressure of 140 Pa at a radio frequency (RF) power of 10 W to reduce the surface oxide layer of the In catalyst and to induce the formation of discrete In droplets. Subsequently, a 7 nm thick a-Si layer was deposited at 110 °C, utilizing a silane flow rate of 4.8 sccm and a chamber pressure of 20 Pa with an RF power of 2 W. Finally, the c-SiNWs were grown under conditions that In droplets were well guided by single-sided step edges and absorbed a-Si precursor film in vacuum at 290 ℃.

***Remnant a-Si layer etching and SiNWs annealing****:* The residual a-Si layer was selectively removed via reactive ion etching (RIE) using CF_4_ plasma at a flow rate of 30 sccm, chamber pressure of 4 Pa, and RF power of 5 W for 4 minutes. Subsequently, the samples underwent annealing at 850 °C for 120 s in an oxygen-rich environment within a rapid-thermal annealed tube furnace.

***GAA FET Fabrication and Characterizations****:* The source/drain region was patterned using electron beam lithography (EBL). The surficial oxide layer on the SiNWs was etched away with BOE, after which a bilayer of Pt/Au (12/55 nm) or Ti/Au (12/55 nm), was deposited via electron beam evaporation (EBE). The Al_2_O_3_ sacrificial layer was subsequently dissolved by immersing in a diluted alkaline solution (2.5% TMAH, Tetramethylammonium Hydroxide), yielding the suspended SiNWs. This was followed by depositing a stacked dielectric layer of 3 nm Al_2_O_3_ and 7 nm HfO_2_ using ALD. Finally, 30 nm of TiN was deposited as the gate electrode through ALD, which can also be replaced with the deposition of 30 nm Al by using magnetron sputtering. Standard electrical measurements were conducted using a Keithley 2636B at ambient temperature.

**S2 Supplementary Figures**


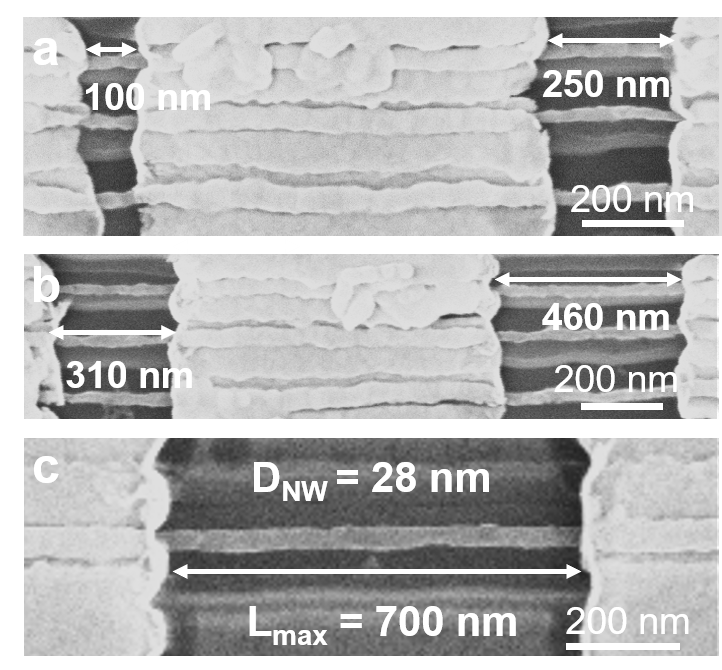


**Fig. S1** SEM images of the suspended SiNWs with suspension length of: **a**) 100 and 250 nm; **b)** 310 and 460 nm; **c)** 700 nm
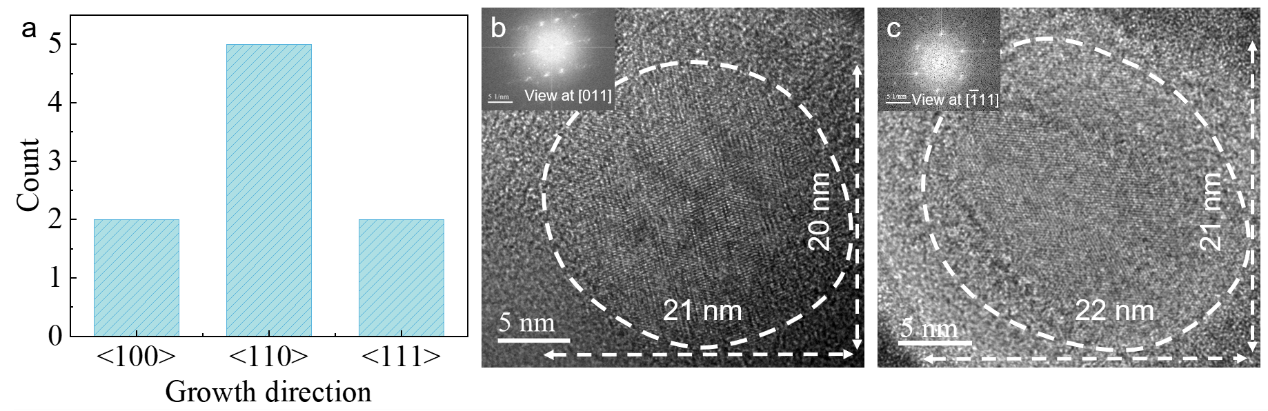


**Fig. S2** **a)** Statistics on growth direction of SiNWs; Enlarged TEM images of the SiNWs with growth orientations of: **b)** [110] and **c)** [-111], the insets show the corresponding FFT patterns


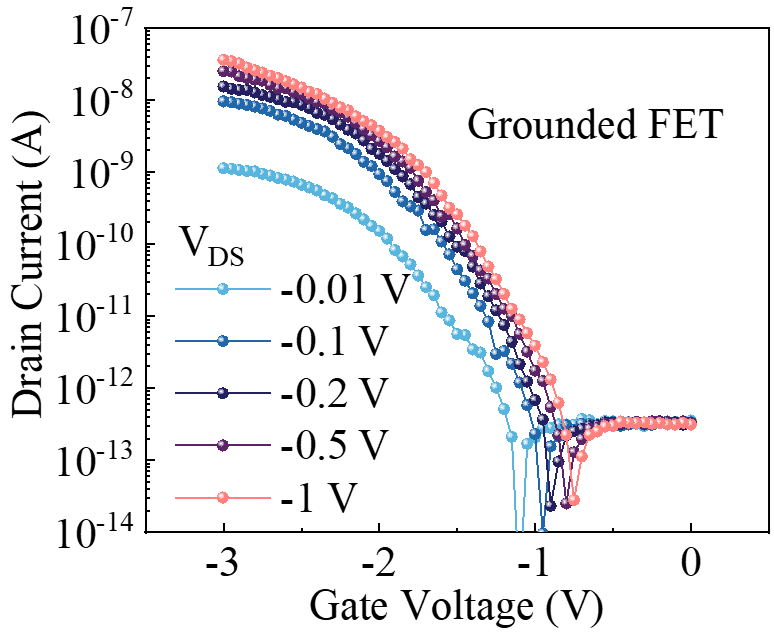


**Fig. S3** Transfer curves of the grounded FET


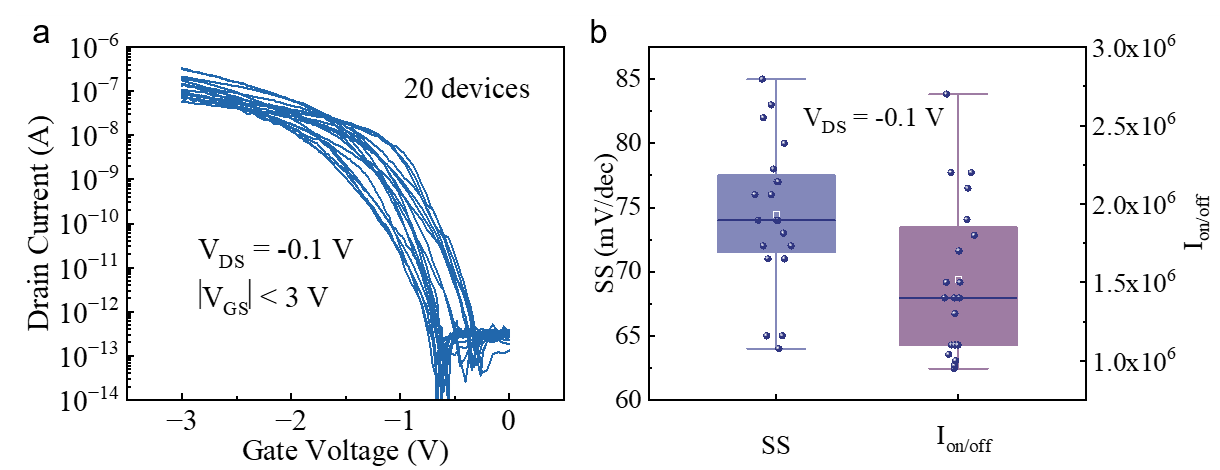


**Fig. S4** **a)** The transfer characteristics of 20 various SiNW GAA-FETs; **b)** Corresponding statistics on the SS value and the I_on/off_ value of the GAA-FETs


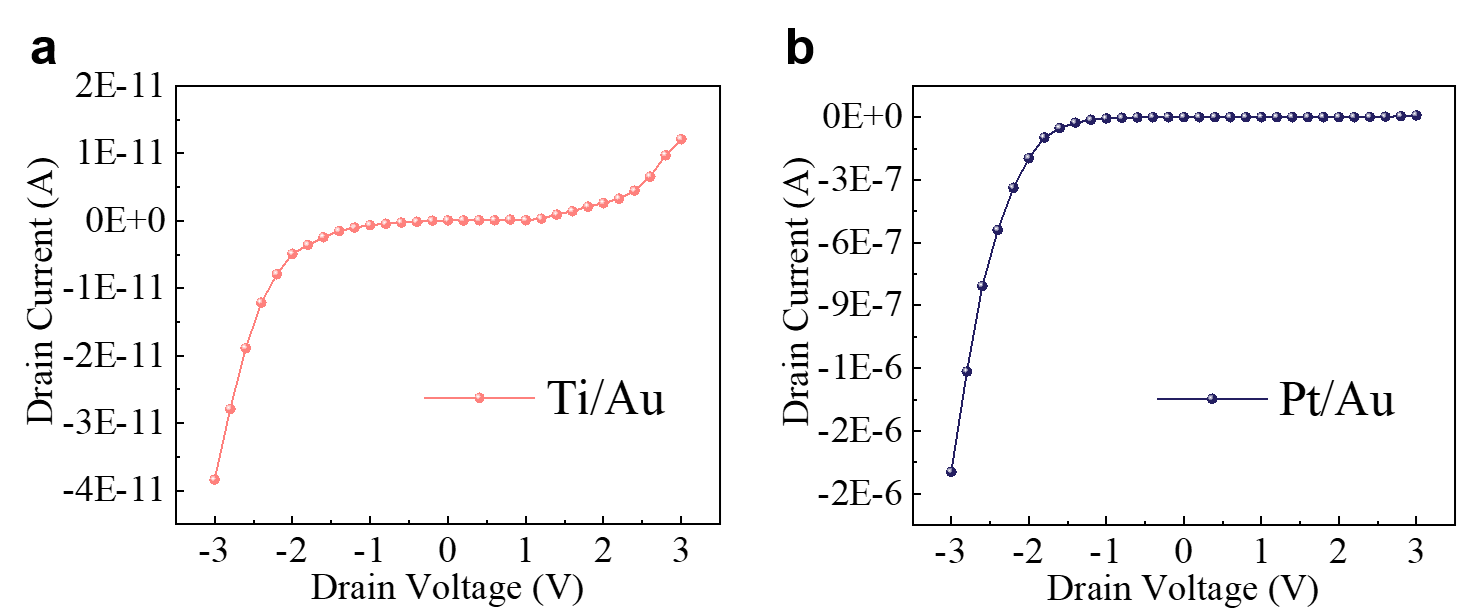


**Fig. S5** Typical I_DS_-V_DS_ curves of GAA FETs with different source/drain contact: **a)** Ti/Au contacted; **b)** Pt/Au contacted

**Table S1** Comparison of our GAA FET to the amorphous oxide semiconductor (AOS), low-dimensional materials, and poly-Si FETs in other literature.

| Channel materials | I_ON_ (A) | I_ON_/I_OFF_ | \|V_D_\| (V) | SS (mV/de**c)** | Refs. |
| --- | --- | --- | --- | --- | --- |
| a-ZTO | 10^-6^ | 10^6^ | 3 | 110 | 2019, [S1] |
| ITO/ZnO | 10^-5^ | 10^7^ | 0.1 | 113 | 2022, [S2] |
| ITO/IGZO | 10^-4^ | 10^9^ | 1 | 92 | 2023, [S3] |
| MoS_2_ | 10^-5^ | 10^7^ | 1 | 80 | 2019, [S4] |
| MoS_2_ | 10^-5^ | 10^7^ | 1 | 70 | 2020, [S5] |
| MoS_2_ | 10^-4^ | 10^8^ | 1 | 220 | 2022, [S6] |
| MoS_2_ | 10^-6^ | 10^5^ | 1 | 117 | 2022, [S7] |
| WSe_2_ | 10^-6^ | 10^7^ | 1 | 142 | 2024, [S8] |
| Poly-Si | 10^-5^ | 10^6^ | 1 | 70 | 2018, [S9] |
| Poly-Si | 10^-8^ | 10^6^ | 0.1 | 75 | 2023, [S10] |
| Poly-Si | 10^-6^ | 10^6^ | 0.05 | 85 | 2023, [S11] |
| IPSLS SiNW | 10^-6^ | 10^7^ | 0.1 | 66 | **This work** |

**Supplementary References**

1. Y. Son, B. Frost, Y. Zhao, R.L. Peterson, Monolithic integration of high-voltage thin-film electronics on low-voltage integrated circuits using a solution process. Nat. Electron. **2**, 540–548 (2019). <https://doi.org/10.1038/s41928-019-0316-0>
2. Q. Li, J. Dong, D. Han, J. Wang, D. Xu et al., Back-end-of-line compatible InSnO/ZnO heterojunction thin-film transistors with high mobility and excellent stability. IEEE Electron Device Lett. **43**, 1251–1254 (2022). <https://doi.org/10.1109/LED.2022.3185099>
3. S. Hooda, M. Lal, C. Chun-Kuei, S.-H. Tsai, E. Zamburg et al., BEOL compatible extremely scaled bilayer ITO/IGZO channel FET with high mobility 106 Cm2/V.s. 2023 7th IEEE Electron Devices Technology & Manufacturing Conference (EDTM). March 7-10, 2023, Seoul, Korea, Republic of. IEEE, (2023)., pp. 1–4.
4. Q. Smets, G. Arutchelvan, J. Jussot, D. Verreck, I. Asselberghs et al., Ultra-scaled MOCVD MoS_2_ MOSFETs with 42nm contact pitch and 250µA/µm drain current. 2019 IEEE International Electron Devices Meeting (IEDM). December 7-11, 2019, San Francisco, CA, USA. IEEE, 23.2.1–23.2.4 (2019).
5. X. Huang, C. Liu, Z. Tang, S. Zeng, L. Liu et al., High drive and low leakage current MBC FET with channel thickness 1.2nm/0.6nm. 2020 IEEE International Electron Devices Meeting (IEDM). December 12-18, 2020. San Francisco, CA, USA. IEEE, 12.1.1-12.1.4 (2020). <https://doi.org/10.1109/iedm13553.2020.9371941>
6. Y.-Y. Chung, B.-J. Chou, C.-F. Hsu, W.-S. Yun, M.-Y. Li et al., First Demonstration of GAA Monolayer-MoS_2_ Nanosheet nFET with 410μA μ m ID 1V VD at 40nm gate length. 2022 International Electron Devices Meeting (IEDM). December 3-7, 2022, San Francisco, CA, USA. IEEE, (2022)., 34.5.1–34.5.4.
7. F. Wu, H. Tian, Y. Shen, Z. Hou, J. Ren et al., Vertical MoS_2_ transistors with sub-1-nm gate lengths. Nature **603**, 259–264 (2022). <https://doi.org/10.1038/s41586-021-04323-3>
8. R. Pendurthi, N.U. Sakib, M.U.K. Sadaf, Z. Zhang, Y. Sun et al., Monolithic three-dimensional integration of complementary two-dimensional field-effect transistors. Nat. Nanotechnol. **19**, 970–977 (2024). <https://doi.org/10.1038/s41565-024-01705-2>
9. C.-C. Yang, T.-Y. Hsieh, P.-T. Huang, K.-N. Chen, W.-C. Wu et al., Location-controlled-grain technique for monolithic 3D BEOL FinFET circuits. 2018 IEEE International Electron Devices Meeting (IEDM). December 1-5, 2018, San Francisco, CA, USA. IEEE, (2018)., 11.3.1–11.3.4.
10. C.-H. Chang, S.-C. Yan, C.-J. Sun, M.-Y. Huang, B.-A. Chen et al., Green laser crystallized poly-Si thin-film transistor and CMOS inverter using Hfo2-Zro2 superlattice gate insulator and microwave annealing for BEOL applications. 2023 Silicon Nanoelectronics Workshop (SNW). June 11-12, 2023, Kyoto, Japan. IEEE, (2023)., 31–32.
11. J.-Y. Ku, J.-M. Yu, D.-H. Wang, D.-H. Jung, J.-K. Han et al., Improved SOI FinFETs performance with low-temperature deuterium annealing. IEEE Trans. Electron Devices **70**, 3958–3962 (2023). <https://doi.org/10.1109/TED.2023.3278626>
